# Letter to the editor regarding ‘Causal relationship between 731 immune cells and the risk of diabetic nephropathy: a two‑sample bidirectional Mendelian randomization study’

**DOI:** 10.1080/0886022X.2024.2431148

**Published:** 2024-11-20

**Authors:** Xiangyun Li, Junhao Chen, Jiansong Wang

**Affiliations:** Kunming Medical University Second Hospital, Kunming Medical University Second Affliated Hospital, Kunming, China

Dear Editor,

We read with great interest the recent article by Song et al. [1] on the bidirectional Mendelian Randomization (MR) study exploring the causal relationship between 731 immune cells and the risk of diabetic nephropathy (DN). The authors utilized two-sample MR and identified several immune cells potentially associated with DN, providing valuable genetic insights into this complex disease. While we appreciate the authors’ efforts and recognize the importance of their findings, we have some concerns about certain aspects of the methodology that we believe could be improved to enhance the robustness and comprehensiveness of the study.

First, in this study, the authors used immune cell exposure data derived from an Italian population within Europe, whereas the outcome dataset (GCST90018832) includes approximately 20% Japanese individuals. This heterogeneity in population raises potential issues, as genetic differences across ethnicities can result in inconsistent SNP effects between populations, thereby weakening the efficacy of genetic instrumental variables. To address this, the authors could consider alternative datasets, such as GCST005895, which contains only European individuals, thus reducing the risk of confounding due to ethnic heterogeneity. Moreover, the choice of validation data is equally critical in MR analysis. The authors selected the FinnGen database as a validation cohort, consisting entirely of Finnish individuals. However, the primary outcome dataset is largely derived from Finnish and British populations, leading to substantial overlap between the primary and validation datasets. In MR analyses, validation cohorts should provide independent verification of the primary results, and thus the validation and primary datasets should originate from different countries or regions to avoid sample overlap. Excessive overlap can result in the validation analysis essentially replicating the findings from the same population, diminishing the true purpose of independent validation. Additionally, the authors utilized the R5 version of the online FinnGen database; however, the FinnGen database has since been updated to the R11 version, which includes more individuals and data, offering a more comprehensive and up-to-date source of validation. Using an outdated version of the database weakens the validity of the validation cohort. To improve the accuracy and reliability of the analysis, we recommend the authors consider using databases that better align with the principles of MR analysis.

Second, the authors identified several immune cells with suggestive associations with the outcome. However, I believe that further application of multivariable Mendelian Randomization (MVMR) would significantly improve the scientific rigor and interpretability of the results [2, 3]. Traditional MR analysis typically establishes a causal relationship between a single exposure and a single outcome, while MVMR allows for the simultaneous assessment of multiple exposures, thereby identifying the factors that independently affect the outcome. Researchers could consider multiple immune cells simultaneously, eliminating false associations that depend on other cell types, and thereby identifying the immune cells that truly have independent effects on the outcome. This approach would also help determine whether the Myeloid Dendritic Cell Absolute Count, after FDR correction, can independently influence the outcome. For example, variants such as rs115805162, rs71632979, rs74341264, and rs56199187 appear as instrumental variables in more than one immune cell (for details, see the original text Supplementary Table 1). In fact, variants like rs71632979, rs74341264, and rs144126567 act as instrumental variables in three different immune cells. Therefore, the MVMR method can reveal the effects of independent immune cells.

Finally, the authors performed a reverse MR analysis, primarily focusing on validating the causal relationship between the immune cells found to be positively associated with DN in the forward MR analysis. While this is a commendable approach that effectively prevents confounding due to bidirectional causality, ruling out the possibility that DN influences immune cell levels, this method is limited by the fact that the authors only analyzed a subset of immune cells identified in the forward MR. A more comprehensive reverse MR analysis of all 731 immune cells would better explore the potential impact of DN on immune cells [4]. Specifically, we recommend a complete reverse MR Analysis of all 731 immune cells, with DN as the exposure point, which not only further confirms the possibility of two-way causality, but also sheds light on whether DN causes elevated or reduced levels of certain immune cells. While such an analysis may be valuable for understanding the pathophysiology of DN, especially as it may help identify the types of immune cells that are regulated during DN progression, it is not necessary. Such an analysis could be valuable for understanding the pathophysiology of DN, as it may help identify immune cell types that are regulated during the progression of DN.

We are grateful for the authors’ work and their insightful conclusions.

## Supplementary Material

Supplemental Material
